# Dynamic and Static Features-Aware Recommendation with Graph Neural Networks

**DOI:** 10.1155/2022/5484119

**Published:** 2022-04-21

**Authors:** Ninghua Sun, Tao Chen, Longya Ran, Wenshan Guo

**Affiliations:** ^1^School of Public Administration, Huazhong University of Science and Technology, Wuhan 430074, China; ^2^Innovation Institute, Huazhong University of Science and Technology, Wuhan 430074, China

## Abstract

Recommender systems are designed to deal with structured and unstructured information and help the user effectively retrieve needed information from the vast number of web pages. Dynamic information of users has been proven useful for learning representations in the recommender system. In this paper, we construct a series of dynamic subgraphs that include the user and item interaction pairs and the temporal information. Then, the dynamic features and the long- and short-term information of users are integrated into the static recommendation model. The proposed model is called dynamic and static features-aware graph recommendation, which can model unstructured graph information and structured tabular data. Particularly, two elaborately designed modules are available: dynamic preference learning and dynamic sequence learning modules. The former uses all user-item interactions and the last dynamic subgraph to model the dynamic interaction preference of the user. The latter captures the dynamic features of users and items by tracking the preference changes of users over time. Extensive experiments on two publicly available datasets show that the proposed model outperforms several compelling state-of-the-art baselines.

## 1. Introduction

The amount of information on the Internet continues to grow rapidly, and determining useful information has become increasingly difficult. Fortunately, the advancement of recommender systems can substantially help people deal with the information overload problem. Collaborative filtering (CF) is one of the most famous methods in recommendation algorithms. Therefore, collaborative learning latent representations of users and items from user-item interactions is an important step in CF-based models. However, poor latent representations of users and items remain the factors limiting further performance.

Therefore, researchers have adopted different methods to capture latent representations. Till now, the most commonly used approach for CF is to learn latent features in the embeddings space generated from the user-item rating matrix, such as matrix factorization [1] and deep learning-based CF [2–4]. Some researchers [5, 6] use a bipartite graph to represent user-item interactions to further enhance the latent representations; hence, the topological features of the graph are introduced through graph neural networks (GNNs) [7]. The underlying assumption in leveraging the bipartite graph as input to obtain effective recommendations is as follows: nodes that are connected can spread information by aggregating their neighbors, thereby potentially contributing to capturing high-order features.

Latent representations obtained from dynamic user-item interactions serve as another method. Traditional CF usually defines a decay function of temporal information [8, 9], such as the exponential decay function *e*^−*ωt*^, to capture these dynamic features, while graph-based CF obtains a series of user-item bipartite graphs based on interaction time [10]. The underlying assumption in using temporal information is that the behaviors of users on items are a dynamic interactive process; consequently, the long- and short-term preferences of users are captured.

It is unclear, however, which of these approaches—static recommendation versus dynamic recommendation—is better for predicting user preference on items. The former ignores that user preference is dynamic, thus changing over time. The latter usually requires more parameters and training time than the static recommendation, which limits its application. Furthermore, the introduction of temporal information may bring additional noise, which can hinder the performance and scalability of the model. Two important problems must be solved to deal with these challenges:

1. How to represent the behaviors of users with a dynamic graph? Temporal information is vital for capturing the dynamic preference of users. To avoid introducing additional noise data, utilizing temporal information data more efficiently should be priorities.
2. How to obtain the dynamic features simply and swiftly? In addition to the interaction pairs of users and items, the dynamic graph also includes side information (e.g., temporal information). However, additional information will introduce an increase in the number of parameters and high computational complexity.

To this end, a simple and effective graph-based algorithm is proposed to introduce dynamic features into a static recommender system called dynamic and static features-aware graph recommendation (DSAGR). Firstly, rather than simply timestamps, the dynamic graph of users and items is constructed based on Takens' time embedding theorem [11] to use temporal information efficiently. This work employs the graph convolution network (GCN) [7] to learn the long- and short-term preferences of users because of the expressive graph-based models. Then, a novel module is proposed, that is, the dynamic sequence learning module, to transform the unstructured dynamic graph to structured sequence data to decrease the dynamic model complexity. In particular, convolutional neural networks (CNNs) [12, 13] are used to capture the dynamic features from the sequence data. Finally, the dynamic features and dynamic preference are integrated to obtain the predictor for each user. Our main contributions are as follows.

1. This work can simply and swiftly capture the dynamic features from the constructed dynamic graph.
2. A novel hybrid model is proposed in this work, which can easily capture the users' dynamic preferences.
3. An offline experiment is performed on real-world datasets. The results show that the proposed model successfully performs the personalized recommendation task.

The rest of the paper is organized as follows.  Section 2 elaborates on relevant research.  Section 3 presents the proposed method, while Section 4 discusses the empirical study on the public datasets. Finally, Section 5 contains the conclusions.

## 2. Related Work

Collaborative filtering- (CF-) based recommendation aims to predict the preference of users and then return top-*N* items of the user interests. Heuristic works, such as item- and user-based models, predict the preferences of users on items based on the *k*-nearest neighbor algorithm [14]. Model-based approaches usually learn the user and item with low-rank latent representations through matrix factorization [1]. The inner product between the two lower-rank vectors is then used to obtain the probability of the user clicking on the item.

Furthermore, deep learning has been shown to be particularly well suited to representation learning tasks [15, 16]. Therefore, many deep learning techniques have recently allowed CF to have expressive representation vectors from the historical behaviors of users, such as the multilayer perceptron (MLP), autoencoder (AE), recurrent neural network (RNN), and CNN. Many researchers often consider the combinations of matrix factorization and deep learning techniques for CF recommendation. MLP-based model Wide&Deep [17] captures linear and nonlinear latent features effectively. NeuMF [2] integrates MLP and MF to model high- and low-order interaction features. Furthermore, AE is used for recommendation tasks. Work [18] employs a denoising recurrent AE network and then generalizes it to the CF setting. RNN has been widely used for recommendation due to its excellent performance in modeling sequential data. The variants of RNN, such as long short-term memory (LSTM) [19]and gated recurrent unit (GRU) networks [20], are often employed in practice to overcome the vanishing gradient problem. For instance, work [21] uses LSTM to model the long- and short-term preferences of users. CNN is also a powerful tool [15]. Work [22] uses two parallel CNNs to learn deep representations of users and items. Work [23] integrates CNN and GRU networks to obtain distributed representations of users and items. These representations are then used to regularize the generation of latent features in matrix factorization.

In the last few years, GNN has been widely recognized as a state-of-the-art approach because of its successful applications in recommendation tasks [24]. GNN can effectively learn the structural representations of nodes by aggregating their neighborhood information. A pooling operation is typically used to output the node embeddings after an aggregation function. Many graph-based models are also proposed by using different aggregation and pooling functions such as GCN [25], GraphSAGE [26], and Graph Isomorphism Network (GIN) [27]. Among these models, the most popular recommendation method is LightGCN [6] coupled with NGCF [5]. LightGCN is an effective simplified version of the NGCF by omitting the transformation mechanism and applying the sum-based pooling layer. Some researchers also consider dynamic representation learning to model data. Work [28] employs matrix perturbation to model the changes in graphs, such as the adjacency matrix. Work [29] constructs the user-item interaction graph dynamically based on the users and items embeddings to improve the diversity of recommendations. Furthermore, many graph-based algorithms [24] have been proposed to enrich the presentation of users and items with other auxiliary information [30, 31]. Therefore, this work tries to introduce dynamic features as side information to improve the recommendation performance.

These graph-based models have verified their superiority for the recommendation task. However, these models mainly focus on constructing static graph-based recommendation models without considering their combinations with dynamic graph features. As far as we know, there is no study to introduce dynamic graph features into a graph-based recommendation framework.

## 3. Proposed Method

**Figure 1:**
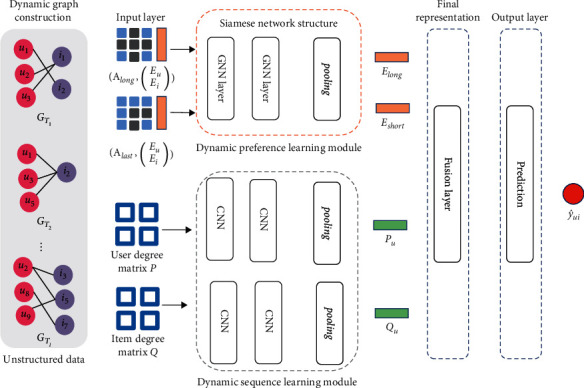
Framework of our model.

In this part, the proposed DSAGR method is presented, and its framework is illustrated in Figure 1. Four components are included in the framework: (1) dynamic graph construction aims to convert the behaviors of users into a dynamic graph; (2) dynamic preference learning module is to learn the long- and short-term preferences of users; (3) dynamic sequence learning module aims to capture the user and item sequence features as side information; and (4) prediction layer is to obtain the predictors.

### 3.1. Dynamic Graph Construction

**Figure 2:**
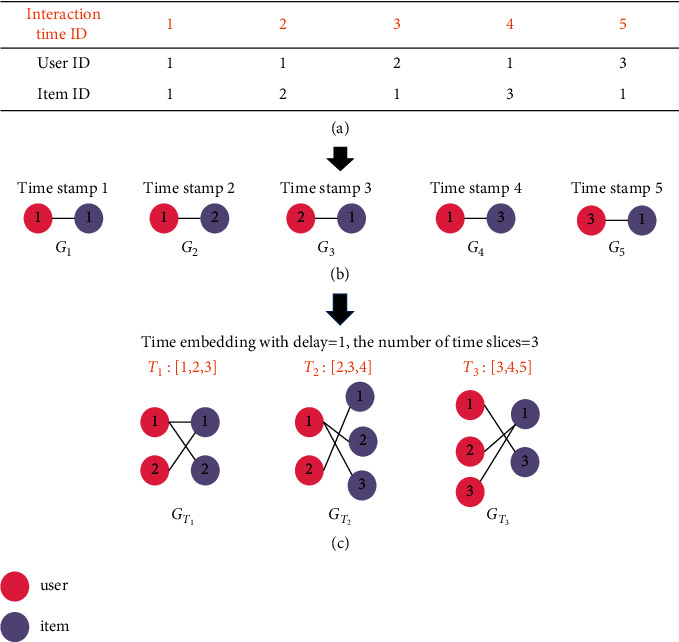
The construction of the dynamic graph. (a) Dataset. (b) The graph of user-item interaction at different time stamps. (c) Dynamic subgraphs.

Given a user set *U*, an item set *I*, and a set of time stamps *T*={*t*_1_, *t*_2_, *t*_3_, ⋯}, the graph of the user-item interaction at the time stamp *t*_1_ can be defined as *G*_*t*_1__=(*U* ∪ *I*, *ℰ*_*t*_1__), where *U* ∪ *I* is the set of nodes, and edge *e* ∈ *ℰ*_*t*_1__ represents the interaction between the user and the item at the time *t*_1_ ∈ *T*. Therefore, the interactions of users and items can be seen as a time series, that is, {*G*_*t*_1__, *G*_*t*_2__, *G*_*t*_3__⋯}.  Figure 2(b) shows different graphs at five different time stamps.

To understand the behaviors of users with the effects of temporal information, several time slices of user-item interactions are generated based on Takens' time embedding theorem [11] using a given delay factor. Considering the following example: given five timestamps [1–5], we assume that the delay factor is equal to 1 and the number of the time slices is 4. Takens' time embedding theorem indicates that the time series is embedded into *R*^2^ vector space as follows: [[1, 2], [2, 3], [3, 4], [4, 5]]. Similarly, the user-item interaction time stamps can also be embedded into the vector space and further be divided into *l* time slices [*T*_1_, *T*_2_, *T*_3_ ⋯ , *T*_*l*_]. Therefore, an interaction graph for each time slice can be obtained as previously mentioned. More formally, the obtained interaction subgraphs are denoted as {*G*_*T*_1__, *G*_*T*_2__, *G*_*T*_3__, ⋯, *G*_*T*_*l*__}.

Figure 2 demonstrates the specific processes.  Figure 2(a) presents the example dataset, which is ranked based on the interaction time in the order from small to large. For the sake of convenience, the interaction time is indicated by numbers 1–5. In (b), the user-item interaction graph at different timestamps can be observed. These user nodes are marked dark red, and item nodes are marked lilac color. In (c), Takens' embedding of temporal information generates three time slices marked by orange color (i.e., *T*_1_: [1–3], *T*_2_: [2–4], and *T*_3_: [3–5]), in which the element is the time ID. The interacted pairs in each time slice constitute a user-item interaction subgraph.

### 3.2. Dynamic Preference Learning Module

The upper part in Figure 1 shows the dynamic preference learning module. In the recommendation task, the long-term preference of users reflects their inherent features and general preference, which can be learned from all interacted items of users. The short-term signals of the user reflect his/her latest preference. Furthermore, many studies [32] use the latest interaction item embedding and the latest timestamp as short-term information but ignore the dependence on historical interactions. The long- and short-term collaboration can be captured effectively by considering the same layer structure with Siamese and information sharing components [33] on all interaction graph *G* and the last subgraph *G*_*T*_*l*__. Siamese networks can naturally introduce inductive biases for invariance modeling because of identical weight-sharing subnetworks. Then, the two graphs can be parameterized using a GNN layer, such as LightGCN [6]. To offer a holistic view of the long-term and short-term collaborative nodes embeddings, we provide the matrix form.

Long-term:

(1) $$\begin{array}{c} \left(\begin{array}{c} L_{u}\\ L_{i} \end{array}\right)=\left(D^{-1/2}AD^{1/2}\right)\left(\begin{array}{c} E_{u}\\ E_{i} \end{array}\right),\\ \left(\begin{array}{c} E_{\text{long},u}\\ E_{\text{long},i} \end{array}\right)=\lambda_{0}\left(\begin{array}{c} E_{u}\\ E_{i} \end{array}\right)+\lambda_{1}\left(\begin{array}{c} L_{u}\\ L_{i} \end{array}\right),\\ A=\left(\begin{array}{cc} M & 0\\ 0 & M^{T} \end{array}\right), \end{array}$$

where *M* ∈ *R*^|*U*|×|*I*|^ is the user-item rating matrix, in which each element *M*_*u*,*i*_ is 1 if the user *u* interacted with the item *i*; otherwise, it is 0. Then, *A* is the adjacency matrix of the graph *G*; *D* is a (|*U*|+|*I*|) × (|*U*|+|*I*|) diagonal matrix, in which each entry *D*_*jj*_ is the number of nonzero entries in the *j*th row vector of the adjacency matrix *A*; *E*_*u*_ ∈ *R*^|*U*|×*d*^, *E*_*i*_ ∈ *R*^|*I*|×*d*^ are the initial weight matrix of users and items, respectively. $\left(\begin{array}{c} E_{u}\\ E_{i} \end{array}\right)$ denotes the concatenation of *E*_*u*_ and *E*_*i*_*λ*_0_, *λ*_1_ ∈ [0,1], are the defined hyperparameters; *E*_long,*u*_ and *E*_long,*i*_ are the final representations of users and items for learning long-term preferences.

Short-term:

(2) $$\begin{array}{c} \left(\begin{array}{c} S_{u}\\ S_{i} \end{array}\right)=\left(D_{\text{last}}^{-1/2}A_{\text{last}}D_{\text{last}}^{1/2}\right)\left(\begin{array}{c} E_{u}\\ E_{i} \end{array}\right),\\ \left(\begin{array}{c} E_{\text{short},u}\\ E_{\text{short},\;i} \end{array}\right)=\lambda_{0}\left(\begin{array}{c} E_{u}\\ E_{i} \end{array}\right)+\lambda_{1}\left(\begin{array}{c} S_{u}\\ S_{i} \end{array}\right), \end{array}$$

where*A*_last_ is the adjacency matrix of the latest subgraph *G*_*T*_*l*__; *D*_last_ is also the diagonal matrix calculated based on *A*_last_. *E*_short,*u*_ and *E*_short,*i*_, respectively, denote the final representation of users and items in the short-term preference learning.

### 3.3. Dynamic Sequence Learning Module

The degree of the graph is shown to be effective for evaluating the popularity of nodes [34, 35]. Therefore, the degree matrixes of users and items are proposed to track the dynamic changes in the user and item nodes in constructed dynamic graph {*G*_*T*_1__, *G*_*T*_2__, *G*_*T*_3__,…, *G*_*T*_*l*__}, respectively. For instance, the degree matrix of items is denoted as *Q*={*q*_1_, *q*_2_, ⋯, *q*_*l*_}, in which element *q*_*k*_ is a |*I*| dimensional vector, its *i*th element *q*_*i*,*k*_ is the number of edges incident to the item *i* in the *k*th subgraph *G*_*T*_*k*__. And the element *q*_*i*,*k*_ means the popularity of item *i* in the time slice *T*_*k*_. Similarly, the user degree matrix is denoted as *P*={*p*_1_, *p*_2_,…, *p*_*l*_}, where *p*_*l*_ ∈ *R*^|*U*|^. Therefore, this study offers a novel means of processing the unstructured graph data and hence may shed light on the task of graph-based recommendation.

The lower part in Figure 1 shows the dynamic sequence features learning modeled by two parallel CNN layers. The input of the module is the obtained user degree matrix and item degree matrix *P* ∈ *R*^|*U*|×*l*^, *Q* ∈ *R*^|*I*|×*l*^. The CNNs generally comprise a set of convolutional and pooling layers in their architectures. In this work, two 1D-convolutional layers and one pooling layer are designed to learn dynamic features. The first and second convolutional layers with a set of {*f*_1_, *f*_2_} filters with the kernel size of *τ*, shared weights *w*_1_ ∈ ℝ^*f*_1_×1×*τ*^, *w*_2_ ∈ ℝ^*f*_2_×*f*_1_×*τ*^, as shown in the following equations.

(3) $$\begin{array}{c} g_{t}=p_{t}*w_{1}, \end{array}$$

(4) $$\begin{array}{c} h_{t}=g_{t}*w_{2},g_{t}\in R^{\left|U\right|\times f_{1}}, \end{array}$$

where *p*_*t*_ ∈ *R*^|*u*|^ is the column vector of user degree matrix *P* ∈ *R*^|*U*|×*l*^, ∗ is the convolution operator, and *h*_*t*_ ∈ *R*^|*U*|×*f*_2_^ denotes a feature matrix for all users. After the 1D-convolutional operation, *l* feature matrixes can be obtained. Inspired by graph-based models [5, 6], the weighted sum operator is designed as the pooling layer and then normalized by the sigmoid function *σ*. The output is shown in the following equation.

(5) $$\begin{array}{c} P_{u}=\sigma \left(h_{1}+h_{2}+\cdots +h_{l}\right). \end{array}$$

Analogously, the items' degree representations are defined as follows.

(6) $$\begin{array}{c} g_{t}^{\prime }=q_{t}*w_{1}^{\prime },\;t=1,2,\ldots ,l,\\ h_{t}^{\prime }=g_{t}^{\prime }*w_{2}^{\prime },\;g_{t}^{\prime }\in R^{\left|I\right|\times f_{1}},t=1,2,\ldots ,l,\\ Q_{i}=\sigma \left(\overset{l}{\underset{t=0}{\sum }}h_{t}^{\prime }\right),h_{t}^{\prime }\in R^{\left|I\right|\times f_{2}}, \end{array}$$

where *q*_*t*_ ∈ *R*^|*I*|^ is the column vector in item degree matrix *Q*, ∗ is the convolution operator, and *w*_1_′ ∈ ℝ^*f*_1_×1×*τ*^, *w*_2_′ ∈ ℝ^*f*_2_×*f*_1_×*τ*^ are shared factors.

### 3.4. Prediction Layer

The embeddings and degree representations of nodes of the user and item are obtained after the dynamic preference learning module and dynamic sequence learning module. Then, a fusion layer is defined to learn the final representations:

(7) $$\begin{array}{c} E_{u}^{*}=\left(E_{\text{long,}u}+E_{\text{short},u},P_{u}\right),E_{i}^{*}=\left(E_{\text{long},i}+E_{\text{short},i},Q_{i}\right), \end{array}$$

where (, ) is the concatenation operator.

Thereafter, we use an inner product on the final embedding of the users and items to predict the recommendation results. The formula is as follows:

(8) $$\begin{array}{c} {\left({\hat{y}}_{u,i}\right)}_{\left|U|\times |I\right|}=E_{u}^{*}{\left(E_{i}^{*}\right)}^{T}. \end{array}$$

### 3.5. Training

In this work, the Bayesian Personalized Ranking (BPR) loss [36] is used, which is a pairwise loss that encourages the prediction of an interacted entry to be higher than its uninteracted counterparts:

(9) $$\begin{array}{c} L_{BPR}=\sum_{\left(u,i,j\right)\varepsilon O}-\ln\;\sigma \left({\hat{y}}_{u,i}-{\hat{y}}_{u,j}\right), \end{array}$$

where *O*={(*u*, *i*, *j*)*|*(*u*, *i*) ∈ *O*^+^, (*u*, *i*) ∈ *O*^−^}, is the dataset in the training process, which consists of interacted pairs set *O*^+^ and uninteracted pairs set *O*^−^. What is more, *L*_2_ regularization is used to optimize the model parameter to prohibit overfitting risk. Therefore, the final objective function in our model is combined by BPR loss and regularization:

(10) $$\begin{array}{c} L_{our}=L_{BPR}+\gamma_{1}{\left\|\Theta_{1}\right\|}_{2}^{2}+\gamma_{2}{\left\|\Theta_{2}\right\|}_{2}^{2}, \end{array}$$

where set Θ_1_={*E*_*u*_, *E*_*i*_} is the set of embedding parameters, Θ_2_={*w*_1_, *w*_2_, *w*_1_′, *w*_2_′} is the set of weights in CNN layers, and *γ*_1_, *γ*_2_ are the hyperparameters to control the regularization. Furthermore, the Adam [37] is used in a minibatch manner to optimize the proposed model.

## 4. Experiments

Empirical results are proposed to evaluate the proposed model. The experiments aim to answer the following research questions:

  RQ1: How does DSAGR perform as compared with state-of-the-art models?
  RQ2: How do dynamic features affect DSAGR?
  RQ3: What are the effects of hyperparameters on the DSAGR model?

### 4.1. Dataset

The dynamic preference learning module in the proposed method requires implicit feedback and temporal information; thus, the proposed model is evaluated on ML_100k and ML_1M movie datasets (https://grouplens.org/datasets/movielens/).  Table 1 presents the statics of the datasets. These datasets have 5-level rating scores, and each user has rated at least 20 movies. The ratings of the datasets are binarized because the proposed model only requires implicit feedback. Specifically, every element in the original rating matrix (scores 1 to 5) is binarized to 1 and 0, where 1 indicates that the rating score is not less than 4, 0 indicates the rating score is less than 4, and no interaction. This work also follows the same settings described in NGCF [5] to select 20% of interaction recodes randomly from each user to represent the test and valid sets and then treat the remaining as the training set.

### 4.2. Experimental Settings

#### 4.2.1. Baselines

The GSAGR model is compared with the following methods:

- Item-based CF (ICF) [38]: ICF is usually a two-step process: (1) determining the similarity set for target items and (2) predicting rating scores based on the most similar items. The rating scores of unseen items for the user are predicted in the second phase according to the weighted average rating of his *k*-nearest neighbor.
- PMF [1]: With the probabilistic matrix factorization (MF) algorithm, this model maps the user-item rating matrix into two low-dimensional matrixes. Then, this algorithm predicts the preference of users by the inner product between the two low-dimensional matrixes.
- DMF [39]: DMF is an MF-based CF method, which obtains the latent features of users and items through deep representation learning, that is, MLP. This method then uses the inner product between the two latent features to predict the preferences of users on items.
- Wide&Deep [17]: Wide&Deep is a famous deep learning recommender system that combines wide linear models and MLP neural network layers to obtain latent representations of users and items.
- NGCF [5]: This work learns the representations of users and items by aggregating the information of high-order neighbors. Specifically, each node obtains the transformed representation of neighbors by propagating embeddings on the bipartite graph structure. NGCF introduces collaborative signals in the pooling layer to enhance high-order latent features learning.
- DGCF [40]: DGCF is an advanced graph-based CF model. This work focuses on the intentions of users for interacting with different items. The implementation of DGCF is based on the NGCF and graph attention network to model different intents of users.

#### 4.2.2. Evaluation Metrics

Unlike the previous studies [17, 39] that perform metrics from sampled uninteracted items, this experiment conducts metrics for all the items that the user has not interacted with. Two widely used evaluation protocols Recall@*N* and NDCG@*N* (normalized discounted cumulative gain) (*N* = 20 by default) are adopted to evaluate the effectiveness of top-*N* recommendation and preference ranking. The specific formula is as follows:

(11) $$\begin{array}{c} \text{Recall}@N=\frac{1}{\left|U\right|}\underset{u}{\sum }\frac{\mathrm{TP}}{\mathrm{TP}+\mathrm{FN}}. \end{array}$$

where TP (i.e., Ture Positive) indicates the number of items in the top-*N* recommendation list that hit the target items and FN (i.e., False Negative) is the number of the positive items in the test set that are falsely identified as the negative items.

(12) $$\begin{array}{c} N\;DC\;G@N=\frac{1}{\left|U\right|}\underset{u}{\sum }\frac{DC\;G@N}{I\;DC\;G@N}, \end{array}$$

where *DC*  *G*@*N*=∑_*i*=1_^*N*^2^*r*_*i*_^ − 1/log_2_(*i*+1); here, *r*_*i*_=1 if the test item is in position *i*, else 0; *I*  *DC*  *G*@*N* indicates the ideal *DC*  *G*@*N* such that the target items are present at the top of the recommendation list.

#### 4.2.3. Parameter Settings

The DSAGR model is implemented in Python under the TensorFlow (https://www.tensorflow.org) framework. For comparison algorithms, the parameter settings are given in the original works of literature. The proposed model uses the following parameter settings: (1) a random normal distribution (the mean and standard deviation are set to 0 and 0.01, resp.) is used to initiate the embedding matrix of users and items. Furthermore, the dimensionality of the embedding matrix is set to 64; (2) the delay factor for dynamic graph construction is set to one three-hundredth of the length of the dataset. (3) GCN and pooling layers with the hyperparameter *λ*_0_=*λ*_1_=1 are used to represent the interaction features of users and items; (3) two GCN layers with 2 and 32 filter factors are used, and the kernel size in each layer is 3; (4) following NGCF [5] and DGCF [40], Adam optimization is used to train the model. The learning rate of the Adam algorithm is 0.0003, which is set by experiments.

### 4.3. Results and Discussion

#### 4.3.1. Performance Comparison (RQ1)

To answer the first research question, the proposed model is compared with six other methods in terms of Recall@*N* and NDCG@*N*. Two of the methods, ICF, and PMF are traditional and are frequently used CF algorithms. DMF and Wide&Deep are deep learning-based CF models. The remaining two, referred to as DGCF and NGCF, are versions of GCN with graph structure.  Table 2 reports the performance for each algorithm. The following observations from this table are presented.

1. This table reveals that DSAGR has achieved the best result on ML_100k and ML_1M datasets. On metrics Recall@*N* and NDCG@*N*, DSAGR has improved the performance by at least 2.72% and 4.81%, respectively. For instance, DSAGR improves the NDCG@*N* over the strongest baselines by 5.798% and 4.81% on ML_100k and ML_1M, respectively. DSAGR can employ the dynamic information simply to provide additional side information for prediction by constructing the degree matrix. Meanwhile, DGCF and NGCF only aggregate the presentations of adjacent nodes in the graph. Significantly, DGCF uses multi-intent-aware graphs but performs worse than the proposed DSAGR model. The reason is that DGCF ignores the dynamic features and the short-term collaborative signals.
2. DGCF, NGCF, DMF, and Wide&Deep achieve better performance than traditional methods PMF and ICF. Therefore, compared with the CF only, the employment of deep learning and GCN is advantageous across the board. Wide&Deep and DMF fail to go beyond NDCG@*N* despite outperforming DGCF in Recall@*N* on the ML_100k dataset, implying that the graph-based methods are more effective than the deep learning methods in modeling the preference of users. In particular, the NGCF model improves Recall@*N* compared with the Wide&Deep model, with enhancements of 2.44%, 5.40%, on ML_100k and ML_1M datasets, respectively. The NGCF model also outperforms the DMF, with at least improvements of 2.68% and 18.61% on Recall@*N* and NDCG@*N*, respectively. Such improvement might be attributed to the GCN module, which captures more complex behavior patterns than MLP.

Furthermore, the experiment is repeated for all methods 10 times. Therefore, the freedom degree of *t*-distribution is 9. Specifically, this experiment accepts the hypothesis that DSAGR achieves better performance than baseline models on the two datasets for significance levels of 0.005. The statistical tests and results for this analysis are shown in Table 3. This table reveals that the method DSAGR successfully enhances the representation of users and items by considering the dynamic features and preferences.

#### 4.3.2. Effect of the Proposed Technologies (RQ2)

The proposed DSAGR is compared with different variants on the ML_100k and ML_1M datasets to investigate the superiority of the key technologies proposed in this work.  Table 4 reports the variant models and their performances. The following findings are presented: DSAGR-L performs better than DSAGR-S, which removes the long-term information. This finding is probably because the preference of users cannot be captured by the short-term information alone. DSAGR-DL, DSAGR-DG, and DSAGR also outperform DSAGR-D and DSAGR-G. This phenomenon proves that the captured dynamic features and short-term information can effectively improve the model's performance. Moreover, DSAGR performs better than GRU-based [20] variant DSAGR-DG and LSTM-based [19] variant DSAGR-DL. This result is probably due to the small length of the row vectors of the dynamic matrix, allowing the CNN to model their dynamic features effectively.

#### 4.3.3. The Sensitivity of Hyperparameters (RQ3)

This work investigates how four hyperparameters, namely, the number of time slices, the filter factors in the first and second CNN layers, and the embedding size, affect DSAGR to examine the effect of the constructed dynamic graph among dynamic preference of users. The experiments on two datasets are conducted, providing similar rules, and only the results on the ML_100k are presented herein.

Inspired by the work [41], the experiment also adopts the orthogonal experimental design (OED) method to get a reasonable combination of these hyper-parameters. Specifically, the number of levels for the four parameters is set as follows: four levels for the time slices {11, 21, 31, 41}; four levels for the filter factors in the first CNN layer {2, 4, 6, 8}; four levels for the filter factors in the second CNN layer {8, 16, 32, 64}; and four levels for the embedding factors {16, 32, 64, 72}. A full-factorial analysis needs 4^4^=256 experiments. Taguchi's method employs the orthogonal arrays to obtain the possible combinations of the hyperparameters from the whole combinations, thus bringing a minimum experimental run and the best estimation of parameters during the execution. In our experiments, the orthogonal array *L*_16_(4^4^) has only 16 experiments, as shown in Table 5. This table shows that DSAGR can achieve better performance by setting time slices as 31, filter factors in the first and second CNN layers as 2 and 32, and embedding factors as 64.

**Figure 3:**
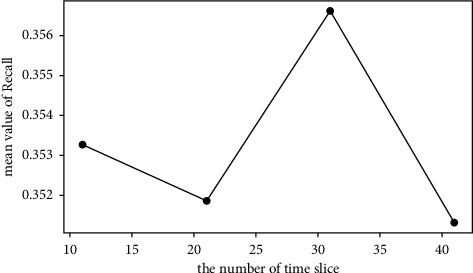
Effect of time slice numbers.

**Figure 4:**
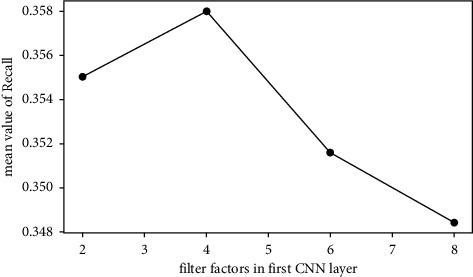
Effect of filter factors in the first CNN layer.

**Figure 5:**
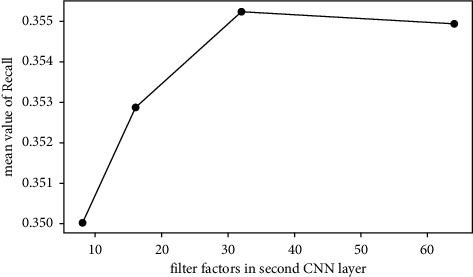
Effect of filter factors in the second CNN layer.

**Figure 6:**
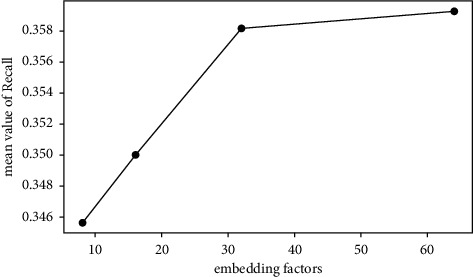
Effect of embedding factors.

The average values of Recall@*N* are used to investigate the effect of each factor. For example, the mean value of the first 4 rows in Table 5 is calculated to investigate the effect of time slice with level 11. The average values of Recall@*N* with different factors are shown in Figures 3–6.

1. *Effect of Time Slice Numbers*. The number of time slices determines the number of dynamic subgraphs and the last subgraph. As shown in Figure 3, DSAGR can achieve the best performance by setting the time slice as 31. Figure 3 shows that when the number of time slices reaches 31, adding more time slices cannot improve the recommendation performance. Also, more time slices will increase the dimension of the row vectors of the degree matrix, and consequently, there will be an increase in the training time taken.
2. *Effect of Filter Factors in CNN*. Figures 4 and 5 show the recommendation performance of different filters in the first and second CNN layers.  Figure 5 reveals that the performance gradually becomes better with the increase of filter factors in the second CNN layer. However, blindly increasing the filter factors does not necessarily improve the performance of DSAGR. This is maybe because that more information is encoded when the filter factors become larger, but it may also bring a little overfitting.
3. *Effect of Embedding Factors*.  Figure 6 illustrates the performance of DSAGR under the different embedding factors. The figure reveals that the performance of the model gradually improves as the dimensionality increases. And the performance tends to be stable when the embedding factors are set as 64.

## 5. Conclusion

In this work, a hybrid recommender system is proposed to capture the dynamic preferences of users and dynamic sequence features. The proposed model, namely, DSAGR, combines GCN and CNN to obtain the latent representations of users and items and then makes a prediction. The dynamic preference is modeled by the long- and short-term interaction graphs of users. The dynamic sequence includes the degree matrixes of users and items captured from the dynamic graph. To our knowledge, this type of modeling using the short-term interaction graph and degree matrixes has not been previously applied to predict users' preferences. The experimental results show that the DSAGR model significantly improves the performance compared with baselines. Considering future work, two feasible avenues are available: (1) The work concentrates on learning the latent representation of users and items via dynamic information. Thus, one direction of further study is to design an effective way to aggregate the long- and short-term representations to a single vector, which is successful in maximizing deep learning. (2) The method of capturing dynamic features provides a new idea to many other unstructured data, such as social networks. It is worth trying to improve the recommendation performance.

## Figures and Tables

**Table 1:** Statistics of the datasets.

Statistics	ML_100k	ML_1M
Number of users	944	6040
Number of items	1683	3952
Number of ratings	100000	1000209

**Table 2:** Performance comparison of different methods.

Datasets				
Metrics	ML_100k		ML_1M	
	Recall@*N*	NDCG@*N*	Recall@*N*	NDCG@*N*
ICF	0.1642	0.2913	0.1504	0.2030
PMF	0.2303	0.3061	0.1943	0.2301
DMF	0.3396	0.3562	0.2215	0.2472
Wide&Deep	0.3402	0.3797	0.2502	0.2613
NGCF	0.3487	0.4225	0.2637	0.2947
DGCF	0.3339	0.4057	0.2973	0.3264
Ours	0.3672	0.4470	0.3054	0.3421

**Table 3:** The *t*-test for paired comparisons in terms of Recall@*N* on the datasets.

ICF	PMF	DMF	Wide&Deep	NGCF	DGCF
*ML_100k*					
337.98	181.01	17.19	14.98	12.88	57.77
<0.005	<0.005	<0.005	<0.005	<0.005	<0.005

*ML_1M*					
305.99	141.38	225.11	73.34	67.96	14.95
<0.005	<0.005	<0.005	<0.005	<0.005	<0.005

**Table 4:** Performance of compared with different variants of DSAGR (“—” indicates DSAGR removes the key technology).

Variants	Dynamic preference		Dynamic feature	ML-100k		ML_1M	
	Long-term	Short-term		Recall@*N*	NDCG@*N*	Recall@*N*	NDCG@*N*
DSAGR-S	—	GCN	CNN	0.36013	0.44148	0.2891	0.3214
DSAGR-L	GCN	—	CNN	0.36370	0.32779	0.3000	0.3325
DSAGR-G	GCN	—	—	0.33217	0.4041	0.2345	0.2911
DSAGR-D	GCN	GCN	—	0.36006	0.44097	0.2798	0.3154
DSAGR-DL	GCN	GCN	LSTM	0.36184	0.44274	0.2921	0.3255
DSAGR-DG	GCN	GCN	GRU	0.36456	0.44921	0.2966	0.3310
DSAGR	GCN	GCN	CNN	0.3672	0.4470	0.3054	0.3421

**Table 5:** Performance of 16 experiments obtained from Taguchi's method.

ID	Time slices	Filter-first CNN layer	Filter-second CNN layer	Embedding factors	Recall@*N*	NDCG@*N*
1	11	2	8	8	0.3422	0.4200
2	11	4	16	16	0.3552	0.4369
3	11	6	32	32	0.3617	0.4420
4	11	8	64	64	0.3540	0.4296
5	21	2	16	32	0.3560	0.4373
6	21	4	8	64	0.3626	0.4412
7	21	6	64	8	0.3451	0.4224
8	21	8	32	16	0.3438	0.4186
9	31	2	32	64	0.3672	0.4470
10	31	4	64	32	0.3660	0.4481
11	31	6	8	16	0.3463	0.4237
12	31	8	16	8	0.3469	0.4259
13	41	2	64	16	0.3547	0.4232
14	41	4	32	8	0.3483	0.41786
15	41	6	16	64	0.3533	0.4217
16	41	8	8	32	0.3491	0.4286

## Data Availability

The data used include ML_100k and ML_1M movie datasets. The movie dataset address is as follows: https://grouplens.org/datasets/movielens/.
